# Needle‐to‐bone safety margin of adrenaline auto‐injectors in Japanese children: Impact of injection site variation

**DOI:** 10.1111/pai.70475

**Published:** 2026-09-16

**Authors:** Chisa Kumagai, Norio Kawamoto, Tomoko Kaneyama, Saori Kadowaki, Yuki Miwa, Tomonori Kadowaki, Minako Kawamoto, Hidenori Ohnishi

**Affiliations:** ^1^ Department of Pediatrics, Graduate School of Medicine Gifu University Gifu Japan; ^2^ Allergy Center Gifu University Hospital Gifu Japan; ^3^ Department of Early Diagnosis and Preventive Medicine for Rare Intractable Pediatric Diseases, Graduate School of Medicine Gifu University Gifu Japan

**Keywords:** adrenaline (epinephrine), anaphylaxis, children, intramuscular injections, ultrasonography

## Abstract

**Background:**

The adequacy of the 12.7‐mm needle length of pressure‐activated adrenaline auto‐injectors (AAIs) for intramuscular administration in Japanese children remains unclear, particularly regarding the injection site within the thigh.

**Methods:**

A single‐center cross‐sectional ultrasonographic study was conducted in children weighing 10–20 kg. Fully compressed skin‐to‐muscle distance (fcSTMD) and skin‐to‐bone distance (fcSTBD) were measured at the distal one‐third of the anterolateral thigh and, when feasible, at the midpoint between the greater trochanter and the lateral femoral epicondyle. Associations with weight, height, thigh circumference, and the Kaup index were assessed, followed by receiver operating characteristic analyses to identify predictors of fcSTBD less than 12.7 mm.

**Results:**

Among 97 children analyzed, the maximum fcSTMD was 8.37 mm at the distal one‐third of the thigh, indicating that a 12.7‐mm needle would reach the muscle in all participants. However, fcSTBD at this site was less than 12.7 mm on one or both sides in 11 children (11.3%). At the midpoint, the maximum fcSTMD was 9.92 mm, and fcSTBD was less than 12.7 mm on one side only in two children (3.7%), both weighing under 12 kg. Thigh circumference and weight predicted fcSTBD less than 12.7 mm at the distal one‐third, with an exploratory weight cutoff around 12 kg.

**Conclusion:**

For Japanese children weighing 10–20 kg, a 12.7‐mm needle in AAIs is generally sufficient to reach the muscle. However, needle‐to‐bone safety varies by injection site, warranting caution in children weighing less than 15 kg. Precise mid‐thigh injection and caregiver education are essential.

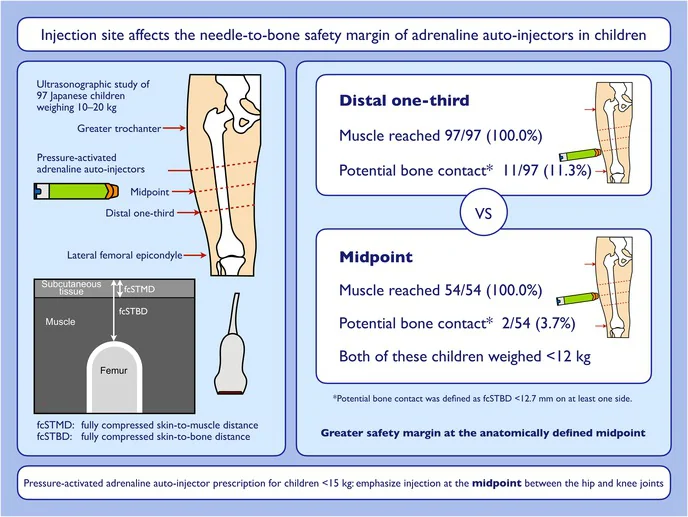

## Graphical abstract

In Japanese children weighing 10–20 kg, the 12.7‐mm needle length exceeded the fully compressed skin‐to‐muscle distance at both locations, indicating muscle penetration. Potential bone contact occurred in 11/97 (11.3%) at the distal one‐third versus 2/54 (3.7%) at the midpoint; all cases occurred below 15 kg, supporting precise midpoint injection.

**Abbreviations**
AAI: adrenaline auto‐injector
AUC: area under the curve
fcSTBD: fully compressed skin‐to‐bone distance
fcSTMD: fully compressed skin‐to‐muscle distance
IQR: interquartile range
ROC: receiver operating characteristic
SD: standard deviation
STBD: skin‐to‐bone distance
STMD: skin‐to‐muscle distance

**Key message**

A 12.7‐mm adrenaline auto‐injector needle generally reached the muscle at the anatomically defined midpoint among Japanese children weighing 10–20 kg, whereas the needle‐to‐bone safety margin was reduced at the distal one‐third of the thigh. These findings support clear instruction to inject at the midpoint between the hip and knee joints, particularly when adrenaline auto‐injectors are prescribed for smaller children.

## 1 INTRODUCTION

Anaphylaxis is a potentially life‐threatening systemic hypersensitivity reaction involving the skin, mucosal tissue, and the respiratory, cardiovascular, and gastrointestinal systems.^1^ Anaphylaxis has various triggers, most commonly foods, medications, and insect stings.^2^, ^3^, ^4^ It often occurs at home and in outdoor settings.^2^ Despite a reported increase in the incidence of food‐induced anaphylaxis, there has been no concomitant increase in anaphylaxis‐related mortality.^3^, ^5^, ^6^ These observations underscore the importance of prompt and appropriate prehospital management of anaphylaxis.

The first‐line treatment for anaphylaxis is intramuscular adrenaline (epinephrine), with adrenaline auto‐injectors (AAIs) playing a crucial role in facilitating prompt administration in emergency situations. Prehospital adrenaline administration is associated with improved clinical outcomes, including reduced biphasic reactions, shorter emergency department stays,^7^ and lower intensive care unit admission rates.^8^ Intranasal adrenaline formulations have recently become available in certain countries for adult and pediatric patients aged 4 years and above weighing at least 15 kg.^9^ However, AAIs remain widely used in the prehospital management of anaphylaxis in pediatric populations. Given that AAIs are often administered by caregivers or other non‐medical personnel in emergency settings, accurate injection technique and precise identification of the injection site are essential for ensuring safe and effective treatment.

Regarding the adequacy of needle length in AAIs among pediatric patients, children weighing less than 15 kg are at risk of unintentional bone contact.^10^, ^11^, ^12^ Moreover, the needle may fail to reach the muscle or penetrate the bone depending on body size and anthropometric characteristics, which may lead to inadequate intramuscular administration, diminished efficacy, and safety concerns in this population. However, these studies were predominantly conducted in North American populations, with the majority of participants being non‐Asian. Consequently, the suitability of the AAI needle length in Asian pediatric populations remains unclear. Specifically, the influence of variations in injection site within the anterolateral thigh, particularly under conditions simulating real‐world AAI administration, on the needle‐to‐bone safety margin in pediatric populations remains unclear.

AAIs are available in several dosages, with most countries predominantly utilizing the 0.15 mg and 0.3 mg formulations.^13^ Notably, the 0.1 mg formulation is currently accessible only in North America, with most countries using the 0.15 mg formulation for pediatric patients.^13^ A needle length of 12.7 mm is typically employed in pressure‐activated AAIs for pediatric patients.^14^ In Japan, an AAI with a dose of 0.15 mg and a needle length of 12.7 mm has been approved for use in children. However, the adequacy of this needle length in ensuring intramuscular injection and maintaining an appropriate needle‐to‐bone safety margin among Japanese pediatric patients remains unclear. Therefore, we aimed to assess the adequacy of a 12.7‐mm needle length for intramuscular thigh injections in Japanese children and to examine how variations in the injection site within the anterolateral thigh influence the needle‐to‐bone safety margin.

## 2 METHODS

### 2.1 Study design and participants

This single‐center, observational, cross‐sectional study was conducted at Gifu University Hospital between March 2017 and March 2026. The study population consisted of children weighing between 10 and 20 kg, including both healthy children and those with food allergies. The exclusion criteria were as follows: underlying medical conditions or physical disabilities affecting lower limb function, including neuromuscular disorders, and local thigh conditions such as abscess, cellulitis, hematoma, lymphedema, edema, or a history of thigh surgery. Furthermore, participants who could not cooperate with the study procedures or were deemed unsuitable for participation by the attending physician were also excluded. The greater trochanter and the lateral femoral epicondyle were identified by palpation, and the distance between them was measured bilaterally. Points at one‐half and one‐third of this distance from the lateral femoral epicondyle toward the greater trochanter were defined as the midpoint and distal one‐third, respectively. The distal one‐third represented the distal boundary of the recommended middle‐third injection region, accounting for real‐world variation in injection‐site localization and allowing comparison with the midpoint. Midpoint measurements were obtained only when tolerated. The study was approved by the Ethics Committee of Gifu University Graduate School of Medicine (No. 28‐517). Written informed consent was obtained from the parents of all participants following a comprehensive explanation of the study procedures.

### 2.2 Ultrasonographic measurement and variable definition

**FIGURE 1:**
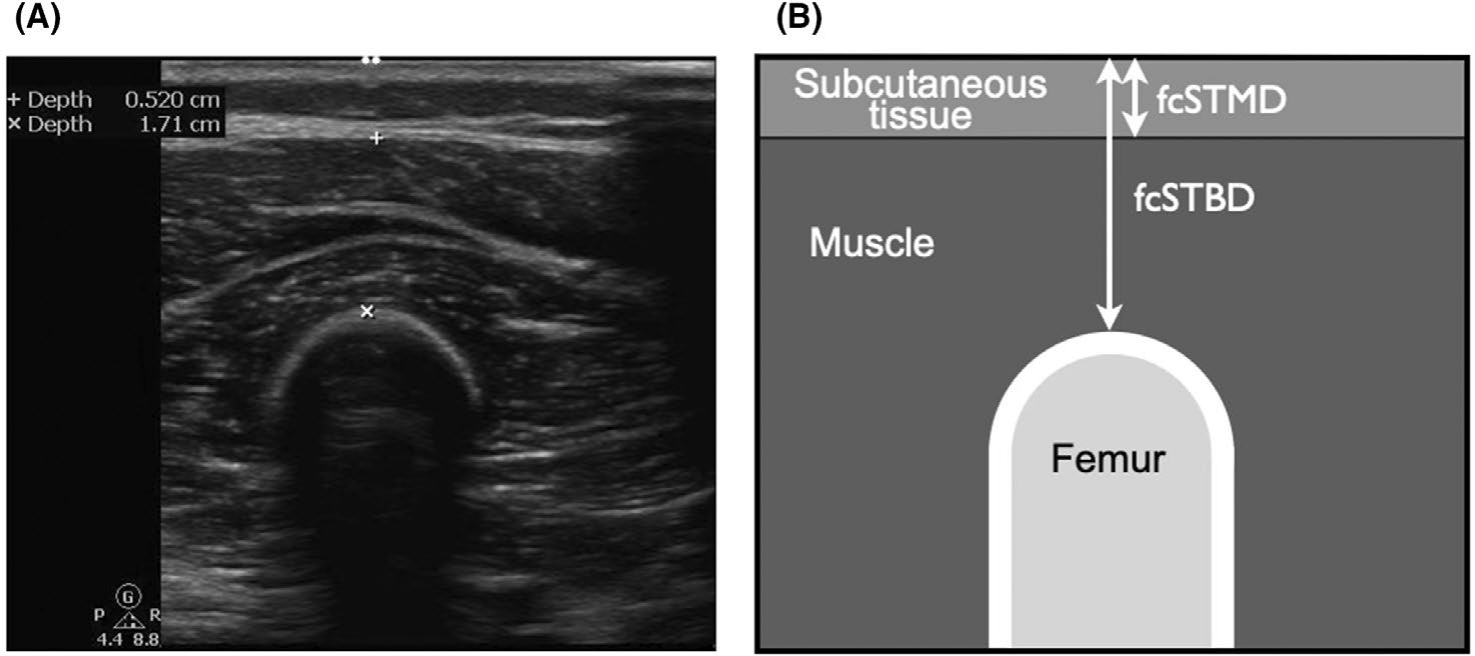
Representative ultrasonographic image and schematic illustration of fully compressed skin‐to‐muscle distance (fcSTMD) and fully compressed skin‐to‐bone distance (fcSTBD). (A) Representative ultrasonographic image of the anterolateral thigh obtained under firm probe pressure to simulate tissue compression during administration of a pressure‐activated adrenaline auto‐injector. (B) Schematic illustration of the measurement of fcSTMD and fcSTBD. fcSTBD, fully compressed skin‐to‐bone distance; fcSTMD, fully compressed skin‐to‐muscle distance.

Ultrasonographic examinations were conducted by four pediatric specialists experienced in the management of anaphylaxis and trained in the use of AAIs. The procedures for measurements, anatomical landmarks, probe orientation, and application of probe pressure were standardized among all operators to ensure consistency in the measurement procedures. Newly participating examiners completed several supervised joint measurement sessions before performing measurements independently. Ultrasonographic measurements of skin‐to‐muscle distance (STMD) and skin‐to‐bone distance (STBD) were performed at the distal one‐third of the anterolateral thigh using a Philips CX50 CompactXtreme ultrasound system equipped with an L12‐3 linear array transducer (Philips Healthcare, Andover, MA, USA). The same ultrasound system and measurement protocol were used throughout the study period. Additional measurements at the midpoint between the greater trochanter and the lateral femoral epicondyle were obtained if tolerated by the patient. All measurements were obtained by applying the transducer perpendicular to the skin until no further reduction in tissue thickness was observed by the operator, which was intended to simulate the tissue deformation that occurs during pressure‐activated AAI administration. Compression force was not measured using a pressure sensor. Measurements obtained under these conditions were defined as fully compressed STMD (fcSTMD) and fully compressed STBD (fcSTBD) (Figure 1). Bilateral measurements were obtained, and the shorter value was used as a conservative estimate of the needle‐to‐bone safety margin. Thigh circumference was bilaterally measured at the midpoint and the distal one‐third of the thigh using a measuring tape, with the mean of the bilateral values at each site used for the corresponding analysis. Height and weight were measured based on standard clinical procedures, and the Kaup index was calculated as weight (kg) divided by the square of height (m).

### 2.3 Statistical analysis

Statistical analyses were conducted to evaluate the relationships of fcSTMD and fcSTBD with anthropometric variables. Continuous variables were presented as median and interquartile range or mean and standard deviation, as appropriate. Among children measured at both sites, paired fcSTMD and fcSTBD values at the distal one‐third and midpoint were compared using the Wilcoxon matched‐pairs signed‐rank test. Associations of fcSTMD and fcSTBD with weight, height, thigh circumference, and the Kaup index were assessed using Spearman's rank correlation coefficients. Scatter plots with fitted lines were generated to aid visualization. To assess the predictive utility of anthropometric measures for an fcSTBD value shorter than 12.7 mm, corresponding to the needle length of a pressure‐activated adrenaline auto‐injector, receiver operating characteristic (ROC) analyses were performed with a threshold of 12.7 mm. The area under the curve (AUC) was calculated for each variable, with exploratory cutoff values estimated using the Youden index. All statistical analyses were performed using GraphPad Prism (version 9.0). Statistical significance was set at a two‐sided *p* value <.05.

### 2.4 Use of generative artificial intelligence

During the preparation of this manuscript, ChatGPT was used to improve readability and language. The authors reviewed and edited all AI‐assisted text and take full responsibility for the content of the manuscript.

## 3 RESULTS

A total of 100 children were initially recruited and provided consent for participation. Of these, 3 were excluded because reliable measurements could not be obtained. Accordingly, 97 children were included in the final analysis. Measurements at the distal one‐third of the thigh were successfully obtained in all participants, whereas measurements at the midpoint between the greater trochanter and the lateral femoral epicondyle were feasible in 54 children. The demographic and anthropometric characteristics of the study population are summarized in Table 1. Overall, 59 boys and 38 girls were included. The median age was 44.0 months (interquartile range [IQR], 33.0 months), with a mean height of 96.4 cm (standard deviation [SD], 11.5 cm) and a median weight of 14.1 kg (IQR, 4.8 kg). The mean Kaup index was 15.6 (SD, 1.4). The distributions of age, height, weight, and Kaup index were comparable between children with and without midpoint measurements (Table 1).

**TABLE 1:** Characteristics of the study participants.

	Total	With midpoint measurements	Without midpoint measurements	*p* value
	*N* = 97	*N* = 54	*N* = 43	
Age, months, median (IQR)	44.0 (33.0)	44.5 (30.5)	44.0 (38.0)	0.4463
Sex, *n* (%)				
Male	59 (60.8)	33 (61.1)	26 (60.5)	>0.9999
Female	38 (39.2)	21 (38.9)	17 (39.5)	
Height, cm, mean (SD)	96.4 (11.5)	97.6 (11.0)	94.9 (12.1)	0.2497
Weight, kg, median (IQR)	14.1 (4.8)	14.5 (4.9)	13.2 (4.5)	0.1005
Kaup index, mean (SD)	15.6 (1.4)	15.7 (1.3)	15.6 (1.6)	0.7918

*Note*: Age and weight were compared using the Mann–Whitney test, sex was compared using Fisher's exact test, and height and Kaup index were compared using Student's *t*‐test.

Abbreviations: IQR, interquartile range; SD, standard deviation.

**FIGURE 2:**
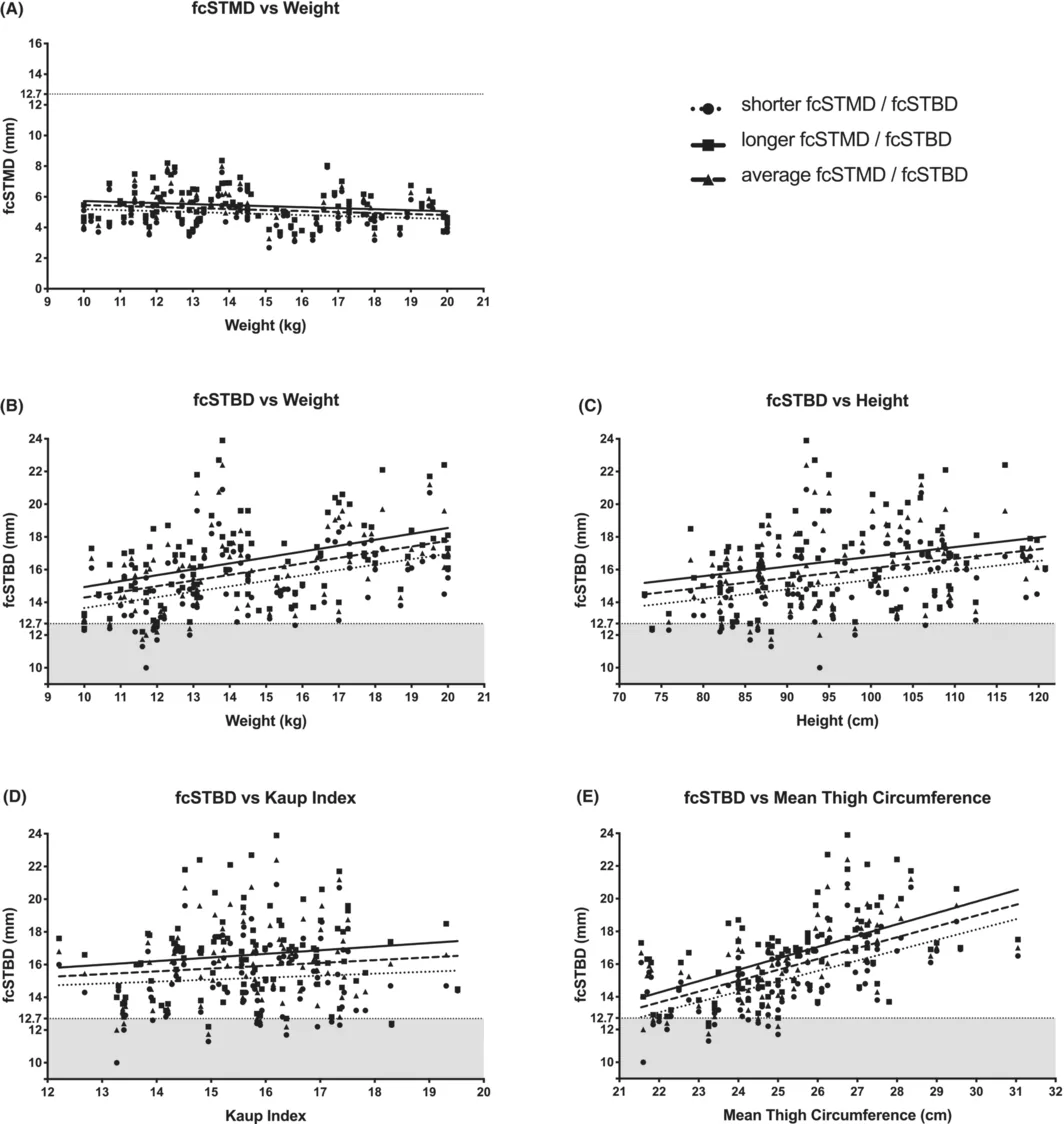
Fully compressed skin‐to‐muscle distance (fcSTMD) and fully compressed skin‐to‐bone distance (fcSTBD) at the distal one‐third of the thigh in children. (A) fcSTMD vs. Weight at the distal one‐third of the anterolateral thigh. (B) fcSTBD vs. Weight at the same site. (C) fcSTBD vs. Height. (D) fcSTBD vs. Kaup Index. (E) fcSTBD vs. Mean Thigh Circumference. Each panel shows a scatter plot with fitted lines indicating the correlations between the respective variables. Both fcSTBD and fcSTMD were measured bilaterally in each participant; the shorter, longer, and average values of each measurement are plotted separately, with corresponding fitted lines for each. fcSTBD, fully compressed skin‐to‐bone distance; fcSTMD, fully compressed skin‐to‐muscle distance.

The distributions of fcSTMD and fcSTBD at the distal one‐third of the thigh are shown in Figure 2, and the median and range of the shorter, longer, and average bilateral values at both measurement locations are provided in Table S1. Figure 2A presents a scatter plot of weight versus fcSTMD. No clear association was observed between these two variables. The maximum fcSTMD observed was 8.37 mm (Table S1), indicating that a needle length of 12.7 mm would reach the muscle layer in all included children. Furthermore, the longer bilateral fcSTMD values were not significantly correlated with weight. Figure 2B–E show scatter plots of fcSTBD at the distal one‐third of the thigh plotted against weight (Figure 2B), height (Figure 2C), Kaup index (Figure 2D), and mean thigh circumference (Figure 2E). The shorter bilateral fcSTBD values showed significant positive correlations with weight (Spearman *r* = 0.49, 95% CI 0.31–0.63, *p* < .0001), height (*r* = 0.35, 95% CI 0.16–0.52, *p* = .0004), and mean thigh circumference (*r* = 0.64, 95% CI 0.50–0.75, *p* < .0001). Among these variables, mean thigh circumference showed the strongest correlation with the shorter fcSTBD values. In contrast, there was no significant correlation between the shorter fcSTBD values and the Kaup index (*r* = 0.08, 95% CI −0.13 to 0.28, *p* = .45). At the distal one‐third of the thigh, the fcSTBD was shorter than 12.7 mm in 11 children (11.3%), including two children (2.1%) with bilateral values shorter than 12.7 mm (Table S1).

**FIGURE 3:**
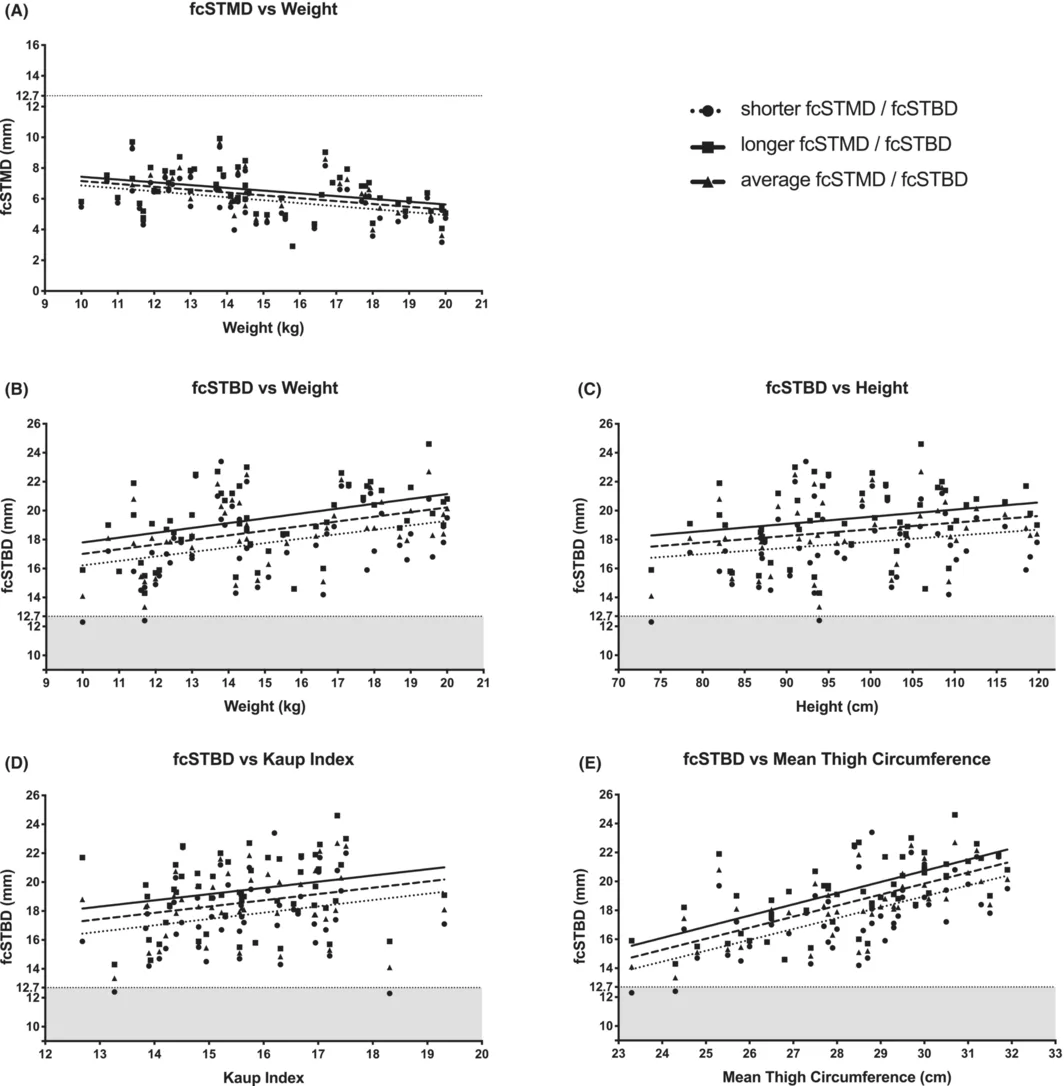
Fully compressed skin‐to‐muscle distance (fcSTMD) and fully compressed skin‐to‐bone distance (fcSTBD) at the midpoint between the greater trochanter and the lateral femoral epicondyle. (A) fcSTMD vs. Weight at the midpoint between the greater trochanter and the lateral femoral epicondyle. (B) fcSTBD vs. Weight at the same site. (C) fcSTBD vs. Height. (D) fcSTBD vs. Kaup Index. (E) fcSTBD vs. Mean Thigh Circumference. Each panel shows a scatter plot with fitted lines indicating the correlations between the respective variables. Both fcSTBD and fcSTMD were measured bilaterally in each participant; the shorter, longer, and average values of each measurement are plotted separately, with corresponding fitted lines for each. fcSTBD, fully compressed skin‐to‐bone distance; fcSTMD, fully compressed skin‐to‐muscle distance.

At the midpoint between the greater trochanter and the lateral femoral epicondyle, the relationship between weight and fcSTMD is shown in Figure 3A. The maximum fcSTMD at this site was 9.92 mm (Table S1), and all measurements were below 12.7 mm, indicating that the needle length of 12.7 mm would reach the muscle layer in all participants at the midpoint. Among the 54 children with measurements at both locations, paired comparisons showed that the shorter, longer, and average bilateral values of both fcSTMD and fcSTBD were significantly greater at the midpoint than at the distal one‐third (all *p* < .0001, data not shown). The relationships of fcSTBD at the midpoint with weight (Figure 3B), height (Figure 3C), Kaup index (Figure 3D), and mean thigh circumference (Figure 3E) are shown. At the midpoint, the fcSTBD was shorter than 12.7 mm on one side only in two children (3.7%), and no child had values shorter than 12.7 mm bilaterally (Table S1). These children were a 17‐month‐old boy (height, 73.9 cm; weight, 10.0 kg; thigh circumference, 23.3 cm; Kaup index, 18.3) and a 50‐month‐old girl (height, 93.9 cm; weight, 11.7 kg; thigh circumference, 24.3 cm; Kaup index, 13.3).

**FIGURE 4:**
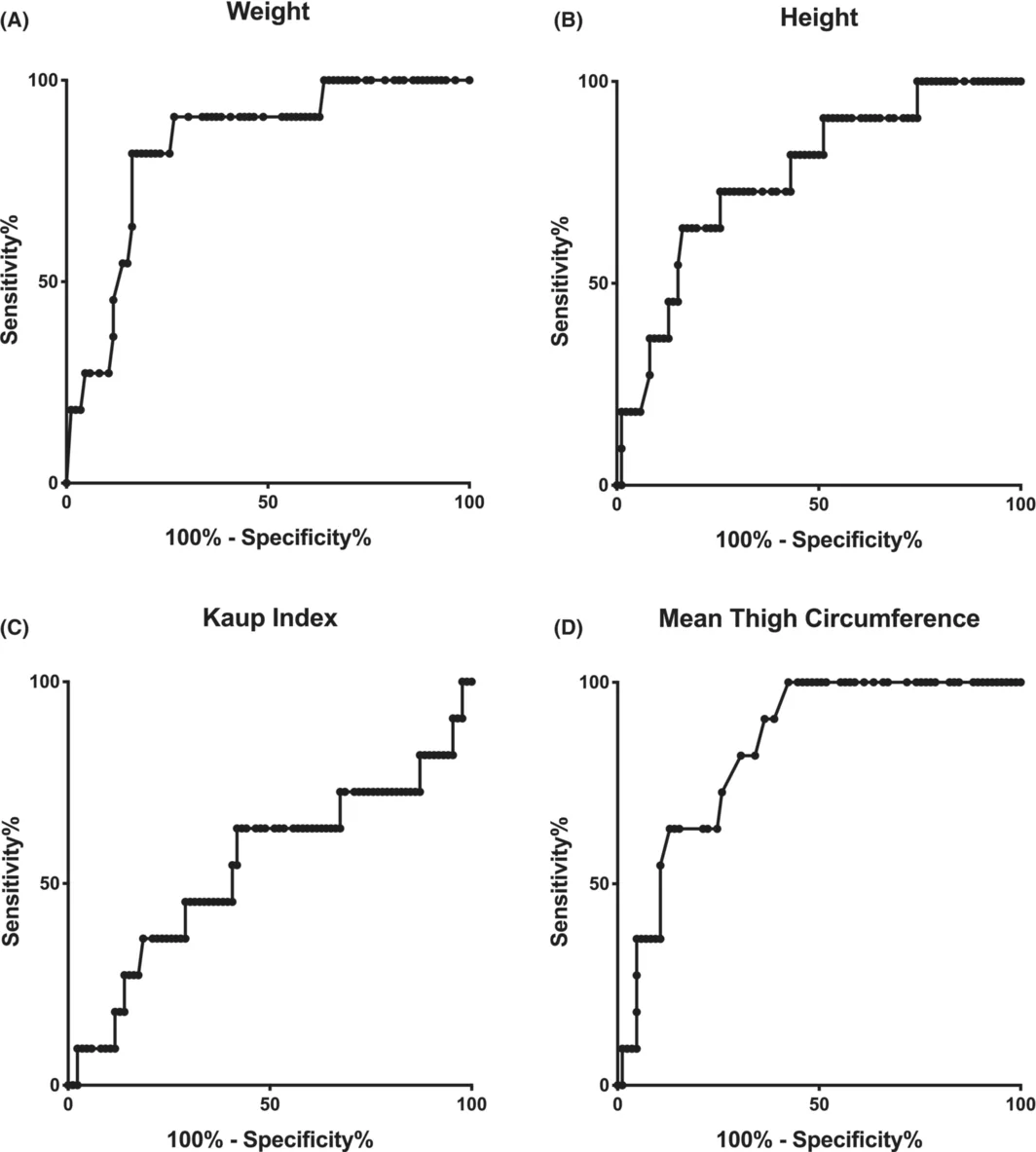
Receiver operating characteristic curves for predictors of fcSTBD less than 12.7 mm at the distal one‐third of the thigh. Weight (A), Height (B), Kaup Index (C), and Mean Thigh Circumference (D) are presented as predictors of fcSTBD less than 12.7 mm. AUC, area under the curve; fcSTBD, fully compressed skin‐to‐bone distance; ROC, receiver operating characteristic.

ROC analyses were performed to assess the discriminative ability of anthropometric measures for an fcSTBD value of less than 12.7 mm at the distal one‐third of the thigh (Table 2 and Figure 4). Weight (Figure 4A) and mean thigh circumference (Figure 4D) demonstrated the highest AUC values, with only minimal differences between them, followed by height (Figure 4B). In contrast, the Kaup index (Figure 4C) showed relatively low discriminative performance. In exploratory analyses using the Youden index, cutoff values were estimated as 25.05 cm for mean thigh circumference, 12.05 kg for weight, and 86.55 cm for height. At the midpoint, ROC analysis was not performed given that only two children had an fcSTBD value shorter than 12.7 mm. Both children weighed less than 12 kg. Regarding laterality, at the distal one‐third of the thigh, the left side showed greater fcSTMD in 58 of 97 children and greater fcSTBD in 50 of 97 children. At the midpoint, the left side showed greater fcSTMD in 33 of 54 children and greater fcSTBD in 27 of 54 children. Overall, no marked laterality was observed.

**TABLE 2:** Receiver operating characteristic analysis for predicting fcSTBD less than 12.7 mm at the distal one‐third of the thigh.

Parameter	AUC (95% CI)	*p* value	Exploratory cutoff based on the Youden index		
			Sensitivity, % (95% CI)	Specificity, % (95% CI)	Threshold
Weight	0.8377 [0.7264–0.9491]	0.0003	81.82 [48.22–97.72]	83.72 [74.20–90.80]	12.05 kg
Height	0.7680 [0.6251–0.9109]	0.0039	63.64 [30.79–89.07]	83.72 [74.20–90.80]	86.55 cm
Kaup index	0.5407 [0.3342–0.7472]	0.6614	63.64 [30.79–89.07]	58.14 [47.01–68.70]	15.83
Thigh circumference	0.8385 [0.7430–0.9340]	0.0003	100.0 [71.51–100.0]	57.65 [46.45–68.30]	25.05 cm

*Note*: Cutoff values were exploratory estimates based on the Youden index.

Abbreviations: AUC, area under the curve; CI, confidence interval; fcSTBD, fully compressed skin‐to‐bone distance.

## 4 DISCUSSION

This study investigated the sufficiency of the AAI needle length for intramuscular injection in the thigh of Japanese children based on ultrasonographic measurements of fcSTMD and fcSTBD. We found that the needle‐to‐bone safety margin varied by injection site, with shorter fcSTBD values observed at the distal one‐third of the thigh than at the midpoint. At the midpoint, the fcSTMD was consistently shorter than the needle length, whereas the fcSTBD exceeded the needle length in nearly all participants. This indicates that intramuscular delivery is generally achieved at this site. Conversely, at the distal one‐third of the thigh, the fcSTMD remained shorter than the needle length across all body sizes, while the fcSTBD was shorter than the needle length in a subset of children with lower weight. Taken together, these findings indicate that although the current needle length is sufficient to reach the muscle, the needle‐to‐bone safety margin is influenced by the injection site.

Ultrasonographic studies have indicated that the AAI needle length may be inadequate for consistent intramuscular delivery in young children and may lead to unintentional contact with bone. A Canadian study involving 100 children weighing less than 15 kg, of whom 78% identified as White, reported that 29% exhibited an STBDmax shorter than the standard 12.7 mm needle length. This included 19% of children weighing between 10 and 14.9 kg and 60% of those weighing less than 10 kg.^11^ Notably, the injection site was described as the anterolateral thigh but was not precisely defined using a specific anatomical landmark or measurement, which may have contributed to variability in the STBDmax even among children within a similar weight category. In the present study, the injection site was defined using specific anatomical landmarks, which facilitated a systematic evaluation of injection‐site–dependent variation in STBD.

Another Canadian study examined infants and toddlers weighing 7.5–15 kg, 81.1% of whom were White, and demonstrated that when measurements were obtained at a precisely defined mid‐thigh site corresponding to the midpoint between the greater trochanter and the lateral femoral epicondyle, the STMD did not exceed the needle length in any participant, indicating that failure to reach the muscle due to excessive subcutaneous tissue was unlikely. However, 43.1% of participants had an STBD measured under applied pressure shorter than 12.7 mm, suggesting a potential risk of unintentional bone contact.^12^ Although this anatomically defined site closely corresponds to the midpoint location evaluated in our study, the broader inclusion of lower‐weight children in the previous cohort may partially explain the higher proportion of short STBD values. Notably, our findings suggest that the principal clinical concern may not lie in modest inter‐study differences at the recommended mid‐thigh site, but rather in the increased risk of insufficient needle–bone distance when the injection site deviates from the midpoint.

Our findings demonstrate that fcSTBD substantially varies across injection sites within the anterolateral thigh, with consistently shorter distances observed at the distal one‐third compared with those at the midpoint. This anatomical difference is clinically relevant given that injection at the distal boundary of the recommended mid‐thigh region is associated with a reduced needle‐to‐bone safety margin during AAI administration. In pediatric anaphylaxis, AAIs are primarily intended for first‐aid use in prehospital settings and are most often administered by caregivers or other non‐medical personnel. Accordingly, the American Academy of Pediatrics emphasizes the importance of training parents and caregivers in anaphylaxis recognition and AAI use, as well as practical preparation in schools and other settings for young people.^15^ In this setting, particularly when an AAI is prescribed to a child weighing less than 15 kg, a simple instruction to identify both the hip and knee joints and inject at their midpoint may improve understanding and recall of the appropriate location. Given that the fcSTBD was shorter at the distal one‐third than at the midpoint in our study, inaccurate localization of the midpoint during real‐world administration could theoretically increase the risk of unintended bone contact, especially in smaller children.

Unintentional AAI–related injuries occur regularly in clinical practice. Indeed, the U.S. Poison Control Centers and the U.S. Food and Drug Administration adverse event reporting system identified more than 15,000 unintentional AAI–related injuries over a 14‐year period.^16^ Although most pediatric injuries involve minor lacerations, there have been reports of needle bending, embedded needles, and difficulty with device removal.^17^, ^18^ Bone contact has been proposed as one possible mechanism in some cases.^18^ Another case report described persistent local pain following AAI use, raising suspicion of unintentional bone penetration, despite the weight and height being considered appropriate for the device.^19^ In this previous study, STBD was measured at the “mid‐thigh,” but the exact anatomical definition of this site, specifically whether it represented the midpoint between the greater trochanter and the lateral femoral epicondyle, was not provided. Notably, intramuscular injection of adrenaline yields more rapid absorption and higher peak plasma concentrations than subcutaneous injection, highlighting the clinical importance of achieving true intramuscular delivery during emergency treatment of anaphylaxis.^20^, ^21^ Taken together, both accurate identification of the injection site and maintenance of an adequate needle‐to‐bone safety margin are critical determinants of safe and effective pediatric AAI use.

Anaphylaxis is predominantly triggered by food allergies; however, its diagnosis is not always correctly established. Moreover, oral food challenge testing is not consistently utilized in routine clinical practice.^22^ Additionally, allergic reactions, including anaphylaxis, frequently occur in community and school environments, where initial recognition and response may be delayed or suboptimal.^23^ Consequently, caregivers of children at risk of anaphylaxis should receive accurate and practical instruction regarding the proper use of AAIs.^15^ The AAI administration technique, including device handling and correct use, is often suboptimal among caregivers and non‐medical personnel, even after prior instruction.^24^, ^25^, ^26^ Educational interventions incorporating repeated hands‐on practice, closed‐loop education, or simulation‐based training can improve correct device handling and user confidence.^23^, ^26^, ^27^ Furthermore, early intramuscular administration of adrenaline is a critical determinant of clinical outcomes in anaphylaxis, with delayed administration being associated with increased morbidity.^15^, ^28^ Accordingly, our findings regarding the anatomical variability offer an additional structural rationale for emphasizing precise injection technique and site recognition during AAI training. Taken together, accurate understanding of the recommended mid‐anterolateral thigh injection site, coupled with adequate compression during administration, may be particularly vital in pediatric populations.

Our exploratory ROC analyses showed comparable discriminative performance for mean thigh circumference and weight, followed by height, whereas the Kaup index showed lower performance. Weight remains the more practical clinical marker because it is generally available before AAI prescription, whereas thigh circumference requires an additional measurement. Notably, both children with fcSTBD values shorter than 12.7 mm at the midpoint weighed less than 12 kg, consistent with the exploratory distal‐site weight cutoff, although 12 kg cannot be considered a definitive clinical threshold. In practice, prescribing an AAI for children weighing less than 15 kg may still be considered based on an overall risk–benefit assessment.^13^, ^29^, ^30^ Therefore, when an AAI is prescribed in this weight group, clear instruction on precise mid‐thigh administration for families and other caregivers is particularly important, consistent with recommendations emphasizing practical caregiver training, recognition of anaphylaxis, and correct administration of an AAI into the thigh.^15^, ^23^, ^24^, ^25^, ^26^, ^27^, ^31^

This study has several limitations. First, the study population consisted exclusively of Japanese children. Although the effect of injection‐site location is likely relevant across populations, the anthropometric cutoff values derived from this cohort may not be directly generalizable to children with different body proportions or growth patterns. Second, full compression was determined qualitatively by the operator without measurement of compression force. Manual transducer compression has not been validated as a proxy for the force profile and tissue deformation produced by an actual pressure‐activated AAI. Third, no formal inter‐rater or intra‐rater reliability assessment was performed, and operator dependence cannot be completely excluded. Fourth, although the same ultrasound system and measurement protocol were used throughout the study period and newly participating examiners underwent supervised training, measurement drift over the 9‐year enrollment period cannot be completely excluded. Fifth, the small number of children with fcSTBD below 12.7 mm limited ROC precision, and the resulting cutoffs remain exploratory pending external validation. Finally, infants weighing less than 10 kg were not evaluated despite their higher likelihood of shorter skin‐to‐bone distances. Therefore, caution is warranted when extrapolating the present findings to this lower‐weight group.

In conclusion, a 12.7‐mm needle length is generally sufficient for intramuscular injection in Japanese children weighing 10–20 kg when administered at the anatomically defined midpoint. Skin‐to‐bone distance varied by injection site and was shorter at the distal one‐third of the thigh than at the midpoint. Weight and mean thigh circumference showed comparable discrimination in exploratory analyses. Given its routine availability, weight remains the more practical clinical marker. These findings highlight that the safe use of AAIs in children depends on accurate mid‐thigh injection and effective caregiver education, especially in smaller children.

## AUTHOR CONTRIBUTIONS

**Chisa Kumagai:** Conceptualization; methodology; data curation; investigation; formal analysis; writing – original draft; resources; writing – review and editing. **Saori Kadowaki:** Resources; writing – review and editing. **Hidenori Ohnishi:** Supervision; writing – review and editing; resources. **Norio Kawamoto:** Conceptualization; methodology; data curation; investigation; formal analysis; resources; writing – original draft; writing – review and editing. **Yuki Miwa:** Methodology; investigation; resources; writing – review and editing. **Minako Kawamoto:** Supervision; writing – review and editing; resources. **Tomoko Kaneyama:** Methodology; investigation; resources; writing – review and editing. **Tomonori Kadowaki:** Conceptualization; methodology; investigation; resources; writing – review and editing.

## CONFLICT OF INTEREST STATEMENT

S.K. is affiliated with the Department of Early Diagnosis and Preventive Medicine for Rare Intractable Pediatric Diseases, Graduate School of Medicine, Gifu University, an endowed department supported by an unrestricted grant from the Gifu Research Center for Public Health. The funding source had no involvement in this study. The other authors declare no conflict of interest related to this study.

## Supporting information


**Table S1:** Bilateral fcSTMD and fcSTBD measurements and frequency of fcSTBD less than 12.7 mm at the distal one‐third and midpoint of the thigh.

## Data Availability

The data that support the findings of this study are available from the corresponding author upon reasonable request.
