# Supplementary material for: Needle‐to‐bone safety margin of adrenaline auto‐injectors in Japanese children: Impact of injection site variation
Source: Pediatr Allergy Immunol. 2026 Sep 16;37(9):e70475. doi: 10.1111/pai.70475 (PMC13580752; doi:10.1111/pai.70475)
Supplement: Supplementary file 1 [file PAI-37-e70475-s001.docx]

|  | **Distal one-third**  **N=97** | **Midpoint**  **N=54** |
| --- | --- | --- |
| Fully compressed skin-to-muscle distance (fcSTMD), mm, median (range) |  |  |
| Shorter fcSTMD | 4.72 (2.68-7.93) | 5.83 (2.91-9.37) |
| Longer fcSTMD | 5.20 (3.53-8.37) | 6.48 (2.91-9.92) |
| Average fcSTMD | 4.94 (3.28-7.99) | 6.27 (2.91-9.56) |
| Fully compressed skin-to-bone distance (fcSTBD), mm, median (range) |  |  |
| Shorter fcSTBD | 14.9 (10.0-20.9) | 17.6 (12.3-23.4) |
| Longer fcSTBD | 16.7 (12.2-23.9) | 19.3 (14.3-31.6) |
| Average fcSTBD | 16.0 (11.8-22.4) | 18.3 (13.4-27.5) |
| fcSTBD <12.7 mm, n (%) |  |  |
| At least one side | 11 (11.3) | 2 (3.7) |
| Both | 2 (2.1) | 0 (0.0) |

**Supplementary Table 1. Bilateral fcSTMD and fcSTBD measurements and frequency of fcSTBD less than 12.7 mm at the distal one-third and midpoint of the thigh**

Data are presented as median (range) or number (%).
